# Electroencephalography in Orthostatic Tremor: A Prospective Study of 30 Patients

**DOI:** 10.5334/tohm.596

**Published:** 2021-05-21

**Authors:** Amy Hellman, John M. Bertoni, Danish E. Bhatti, Najib Murr, Diego T. Russotto

**Affiliations:** 1 Department of Neurological Sciences, University of Nebraska Medical Center, 988440 Nebraska Medical Center, Omaha, NE 68198-8440, US; 2 Geitaoui Hospital, Achrafieh, Beirut, Lebanon

**Keywords:** Tremor, EEG, Movement Disorders, Orthostatic tremor, OT Pathophysiology

## Abstract

**Background:**

Orthostatic tremor (OT) is characterized by a sensation of instability while standing, associated with high frequency (1318 Hz) tremor in the legs. Small retrospective series have reported electroencephalography (EEG) findings in OT with discordant results.

**Methods:**

We prospectively enrolled 30 OT subjects. Mean age = 68.3 (range 5487) with mean disease duration 16.3 years (range 444). A modified 1020 system EEG recording with additional midline electrodes was obtained. EMG electrodes were placed on quadricep muscles. EEG recording was performed at rest, during sleep and while standing unassisted.

**Results:**

In all subjects, EEG showed normal background, normal drowsiness and/or stage 2 sleep, and normal responses to hyperventilation and photic stimulation. These normal results persisted during stance. EEG abnormalities were found in 3 subjects (anterior-mid temporal slow activity), but were not position-dependent and were judged unlikely to be related to OT. Tremor artifact while standing was noted in all subjects, however it was measurable in 26 with frequency in the OT range in 25. When compared with EMG, the average difference in frequency was small at 1.2 Hz (range 0.52.5, p 0.46). Visual EEG analysis in OT patients did not reveal electrographic abnormalities even upon standing unassisted.

**Discussion:**

EEG was normal on this prospective, relatively large OT series. Clinicians interpreting video-EEGs should be aware of the OT artifact that can be seen in EEG and EKG leads mostly while standing.

## Introduction

Orthostatic tremor (OT) is a syndrome characterized by a sensation of instability associated with a very high frequency (1318 Hz) tremor in the legs while standing [1]. The main symptom is the instability, which can be expressed by the patients as a sensation of impending falling, inability to stand, or other symptoms, which typically disappear upon sitting and at least improve while walking or leaning on a firm surface. Although the patients rarely fall, a blinded study showed that OT patients tend to have abnormal Bergs, Tinettis and other balance scales, as it would be expected for this disease that primarily affects the patients perception of truncal stability [2]. While OT can start at any age, its onset is more common in the 40s to 50s. About two-thirds of patients are females.

Although in the past OT has been thought to be etiologically associated with Parkinson Disease and Essential Tremor, it seems clear now that OT is an entity of its own [3,4]. Primary Orthostatic Tremor is seen in the absence of other neurologic abnormalities. However, secondary OT has been thought to present in association with other central or peripheral nervous system conditions.

The pathogenesis of OT is incompletely understood, although current science supports a central oscillator localized within the posterior fossa [5], but few prior studies have shown EEG abnormalities in OT patients [6]. The presence of EEG abnormalities would point toward a cortical localization. Cortical involvement has also been postulated in the past supported by transcranial magnetic stimulation (TMS) studies [7] and the reported response to antiepileptic medications [8,9,10,11,6,12,13], albeit inconsistently with conflicting results [14,15,3,16]. If cortical involvement would be part of the pathophysiology of OT, then EEG could show abnormalities and become useful in the patients evaluations. There have been several reports of normal electroencephalogram (EEG) findings in OT patients, but in a large retrospective review of 19 patients, abnormal fast midline discharges were reported in the EEGs of 5 out of 19 patients with OT [6]. These findings have not been replicated or explained. The presence of EEG abnormalities in OT will be very meaningful for understanding the pathophysiology of OT and expanding research avenues for this disease. We thus aimed to ascertain the presence of abnormalities on EEG recordings in subjects with OT in a prospective fashion including an assessment while standing.

## Methods

This protocol is part of the University of Nebraska Medical Center Orthostatic Tremor study, a large, prospective, multi-faceted study that has collected longitudinal data from a cohort of OT patients from USA, Canada, Europe and Australia. Subjects with previously diagnosed OT were prospectively enrolled in the study after obtaining informed consent as approved by the IRB at the University of Nebraska Medical Center. Baseline epidemiological and clinical data was collected using an intake form. Confirmatory surface EMG was previously available (through our database) in 18 subjects, and was obtained in the 12 remaining patients. Subjects were included if the diagnosis of Primary Orthostatic Tremor was confirmed by one of our Movement Disorder specialists. Subjects were excluded if they had history of vestibular disease, high risk of falling, or other causes of instability or ataxia. Each subject underwent a 45 minute EEG recording using a modified 1020 system which included standard electrodes and additional midline electrodes Fpz, F1, F2, C1, C2, P1, P2, and Oz for better coverage of central and frontal areas in order to compare our results to previous studies which focused on these areas. In addition, an EMG electrode was placed on the quadriceps muscles to record tremor while standing. EEG recordings were done with subjects lying down while awake and asleep, with standard inciting procedures (photic stimulation, hyperventilation), while standing and while walking short steps. This data was digitally recorded (Xltech EEG system) in a standard hospital EEG databank and analyzed by a board-certified epileptologist. Only basic descriptive statistics were necessary to analyze the data.

## Results

The subjects were mostly female (28/30) with an average age of 68.3 years (range 5487). The average duration of OT was 16.3 years (range 444). None of the patients had a history of epilepsy. None of the patients were using anti-epileptics at the time of the recording except two patients who were taking gabapentin (less than 300 mg a day) and one patient who was using clonazepam (0.5 mg per day), all at very low doses for their OT.

The EEG recordings of all 30 subjects showed normal background with an average frequency for posterior dominant rhythm of 10.22 Hz (range 9.512 Hz). EEG of drowsiness was seen in 29 subjects and stage 2 sleep was reached in 14 subjects. The subjects could decide whether or not to participate in hyperventilation and photic stimulation. Hyperventilation was performed in 26 subjects and showed no abnormalities in all 26. Photic stimulation was performed in 29 subjects and posterior driving response was seen in all 29.

EEG abnormalities were seen in only 3 subjects and consisted of medium voltage, intermittent, irregular delta activity in the anterior mid-temporal region, 2 on the right and 1 on the left. These abnormalities were not associated with standing or tremor.

**Figure 1:**
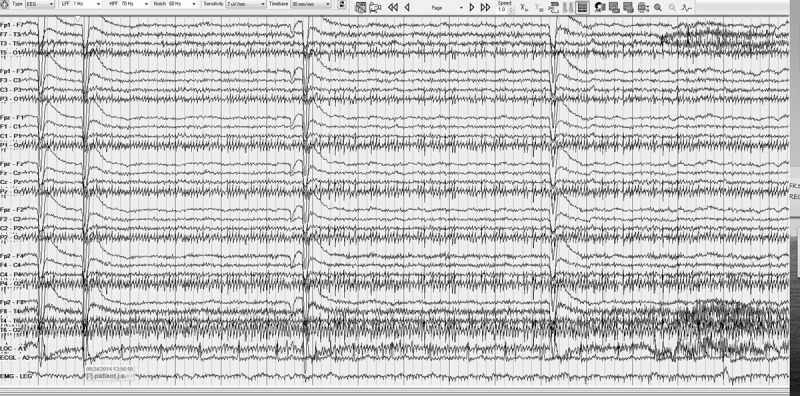
EEG artifact. EEG recording showing tremor artifact in midline and posterior leads.

**Figure 2:**
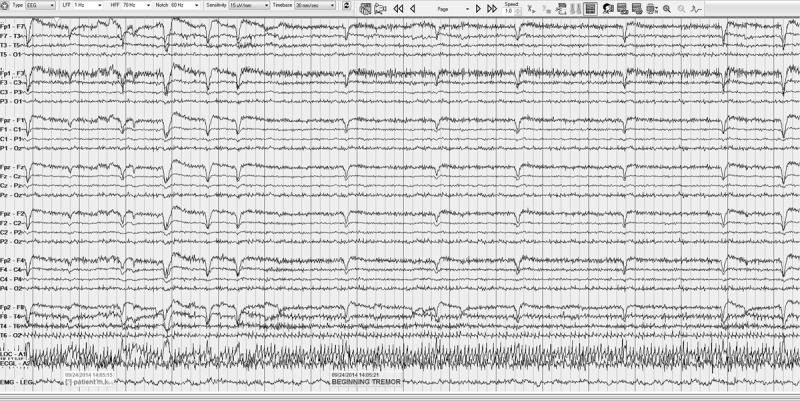
EKG artifact. EEG recording showing tremor artifact in EKG lead.

Tremor artifact was noted in EEG (most often in posterior leads) or EKG electrodes in all subjects (See ***Figures 1*** and ***2***). This was measurable in the EEGs of 26 subjects where the frequency was visually calculated, using a measurement feature built into the EEG software, by the blinded reviewer (NM). The frequency was measured to be in the OT range of 1318.5 Hz (mode 15.5 Hz) in all except for one recording which showed a tremor of 7.3 Hz. This frequency was compared to that found on the 12 subjects who underwent EMG concurrently, for whom recently-measured tremor frequency was available. One subject with low frequency artifact of 7.3 Hz on EEG had a fast OT tremor of 17.5 Hz on EMG. The remaining 11 subjects had very similar tremor frequency in EMG and EEG with an average difference of 1.2 Hz (range 0.52.5). This difference was not statistically significant (p = 0.46). (See ***Table 1***) Tremor artifact was noted in the EKG leads of 3 subjects.

**Table 1:** EEG Results.


SUBJECT	EEG RESULTS	ARTIFACT	TREMOR FREQUENCY ON EMG

1	Normal	15 Hz in posterior electrodes on EEG	15 Hz

2	Normal	15 Hz in occipital electrodes on EEG	No EMG

3	Normal	17.6 Hz in EKG	No EMG

4	Normal	17 Hz in inferior orbital electrode on EEG, also seen in frontal, occipital and temporal electrodes	No EMG

5	Generalized fast activity of low voltage which could be consistent with generalized medications effect	15.6 Hz in infraorbital electrode on EEG	15.6 Hz

6	Normal	Present in bilateral temporal regions on EEG but not able to be measured	15.5 Hz

7	Normal	Present in temporal regions on EEG but frequency did not match (exact frequency not reported)	18 Hz

8	Normal	None noted	Could not be measured

9	Normal	16.5 Hz in T3 and T5 electrodes on EEG and EKG	No EMG

10	Normal	Present in occipital regions on EEG	17 Hz

11	Normal	15 Hz in Oz electrode on EEG	No EMG

12	Normal	13.2 Hz in Oz electrode on EEG	13.2 Hz

13	Normal	15 Hz in T5 and T3 electrodes on EEG while sitting and other midline and central electrodes when standing	No EMG

14	Left anterior mid-temporal slow activity	Could not be measured	No EMG

15	Right anterior mid-temporal slow activity	18 Hz in occipital electrodes on EEG	No EMG

16	Normal	14 Hz in occipital electrodes on EEG	No EMG

17	Normal	Present in occipital electrodes but not able to be measured	15.5 Hz

18	Normal	Could not be measured	Could not be measured

19	Normal	16 Hz in Oz electrode on EEG	No EMG

20	Normal	None	15.5 Hz

21	Normal	14.5 Hz in posterior electrodes on EEG	14.5 Hz

22	Normal	16.5 Hz in Oz and O2 electrodes on EEG	16.5 Hz

23	Normal	14 Hz in infraorbital electrode on EEG	No EMG

24	Normal	18.5 Hz in EKG	No EMG

25	Normal	15.516 Hz in Oz, O1, O2, T5, and T6 electrodes on EEG	No EMG

26	Normal	Present in bilateral temporal, midline, and posterior head leads on EEG but could not be measured	Could not be measured

27	Normal	15.5 Hz in occipital electrodes on EEG	Could not be measured

28	Normal	16 Hz in O1, O2, Oz, and T4 electrodes on EEG	16 Hz

29	Right anterior mid-temporal slow activity	7.3 Hz in O1 and Oz electrodes on EEG	7.3 Hz

30	Normal	Present in EEG but could not be measured	15.5 Hz


## Discussion

There is discordance among studies as to whether there are measurable EEG abnormalities in OT patients. It is also unclear if the nature of such alterations, when present, is due to cortically-recorded rhythms or abnormalities, or the pure result of muscle artifact. However, the presence of EEG abnormalities could be of extreme importance in localizing the OT oscillator, hence the importance of this topic.

Few studies have reported electroencephalographic (EEG) findings in OT, though cortical origin of OT was historically considered and studied in detail. The first clear report of EEG abnormalities in OT was in a retrospective study by McManis et al who reported EEG findings in 19 subjects with OT using the 1020 system and extra midline leads (FPz-Fz, Fz-Cz, Cz-Pz, Pz- Oz). Five of these 19 patients showed abnormal fast 1424 Hz midline discharges greatest at Cz and without lateral spread. Only 2 of these 5 patients had worsening of discharges upon standing. The remaining 14 patients had normal EEG findings [6]. The authors claimed the midline discharges were not due to movement or muscle artifact. Given the lack of detailed explanation of the EEG findings and other limitations, the nature of such abnormalities remained unclear. A more recent chart review study by Hassan et al, reported midline fast rhythm while standing on the EEG of 11 of their 51 patients. The frequency was recorded on only 5 of these patients and was either similar or equal to the EMG frequency [17].

However, a number of authors have previously published negative EEG recordings in OT patients. In one of the few randomized placebo controlled trials in OT using gabapentin (an antiepileptic agent), Evidente et al reported the use of EEG with a 1020 system and standard ear-reference montage that included Cz. Multichannel surface EMG recordings were made, and recordings were done at rest, while standing, walking in place, leaning against a wall, doing the finger-to-nose maneuver, with arms outstretched and with feet outstretched. No actual baseline or post-treatment EEG data was reported in any details, but a note was made that no EEG changes were seen [10]. Many case reports have noted normal EEG recording in lying and standing position [18,19,14] and during periods of leg tremulousness [20].

Cortical involvement in OT has been postulated in the past, but studies have produced conflicting (largely negative) results. One study comparing hand motor cortex stimulation in OT versus controls found only contralateral responses in subjects with OT while bilateral motor responses were seen in healthy subjects [21]. The presence of coherent tremor in cranial muscles noted during isometric contraction despite different body postures was reported as a suggestion for a supra-spinal generator and linking mechanism [21]. Scientists have shown in the past that the OT tremor phase could be reset using TMS of the motor cortex in the leg area [7]. However, another group attempting resetting of OT tremor with TMS in three patients showed no effect. In addition, they looked for effect of static head positioning and found no change. They suggested that the motor cortex is likely not a site or modulator of OT and that static vestibular input does not modify OT [22].

More recently, Schberl et al, have evaluated PET studies in OT patients and revealed the involvement of a cerebellothalamocortical tremor network which still does not identify a single tremor oscillator [23]. Guridi et al, reported PET with EEG recording with back averaging that revealed what they called a cortical rhythmic activity that was phase locked with tremors recorded from the tibialis anterior muscles [24]. Despite the fact that both activities are phase locked, the nature of such activity remains unclear. One could argue that the EEG activity could still represent an OT activity of the scalp muscles, reflecting the earlier onset of the tremors on the scalp. Also, this could be a sensory loop of the tremor coming back to the cortex (as in the chicken-and-egg diatribe of PET-based studies). Since this activity has always been reported in the midline electrodes on surface EEG, performing A MEG scan in patients who have OT and correlating the meg activity with the lower limb EMG activity might theoretically help determining the presence or absence of such midline cortical activity. However, technical difficulties, including but not limited to patient positioning and posture during MEG recording, constitute a main challenge to performing such evaluations.

The possibility of cortical involvement in OT was further suggested by the response of OT to some antiepileptic medications, however data is incomplete and conflicting in this area as well. Additionally, antiepileptic medications have been effective in treating movement disorders of non-cortical origin. Some studies initially reported partial success with primidone alone or as an adjunct to clonazepam [8,9,6,25], but other cases have described no benefits to primidone [3,10,14]. Phenobarbital has been reported to provide benefit in some patients with OT [19,3], yet other studies found no benefits with phenobarbital [10,15]. Some cases and reviews have described benefits to valproate [10,6,26]. Still other studies have noted no benefit [3,15]. Only gabapentin and levetiracetam have been studied in placebo-controlled, randomized clinical trials. Levetriacetam failed to provide benefit in a cross-over study with 12 OT patients with doses up to 3000 miligrams (mg) twice daily [16]. Studies of gabapentin in OT, however, have yielded more positive results. Three separate, small, placebo-controlled trials have shown reduction in tremor in most patients treated with gabapentin [10,13].

We report here the first prospective, relatively large EEG study in OT patients. Visual EEG analysis in OT patients did not reveal consistent electrographic abnormalities, even upon standing unassisted. This is in contrast to previous retrospective studies which identified midline fast rhythm in some subjects with OT. The prospective design of the present study allowed the epileptologist to be present during the EEG recording with most of the subjects, allowing him to perform the tasks himself and adjust the EEG parameters as needed to clarify results. This is why our results differ from previous studies. We believe that the EEG abnormalities that we found in 3 subjects are unlikely to be related to OT, as they were not consistent between subjects and did not persist upon standing. Tremor artifact, however, was detected in the EEG of almost every subject, mostly with very consistent frequencies. Adjustment of frequency filters allows for good distinction between tremor and underlying cortical activity, so the tremor artifact does not mask all underlying cortical activity. We recommend using midline leads when performing routine EEGs in patient with OT as the frequency of the tremor was most appreciated in the midline electodes. Our EEG findings suggest normal cortical function in OT. Of note, we also found artifacts on the EKG lead within EEG in 3 patients, consistent with one previous case report of EKG artifact in OT [27]. However, we did not perform full 12 lead EKGs in our patients and cannot comment further on this artifact.

Our study has some limitations. Due to the limitations of our equipment, we were not able to perform EEG back-averaging that could have helped depicting cortical activities missed on visual EEG analysis. Focal cortical discharges can be missed on routine scalp EEG. Furthermore, not all EEG abnormalities are specific to cortical lesions but may be found with subcortical pathology [28]. So while our normal EEG findings support normal cortical function, we cannot definitively rule out cortical pathology in OT from this one study. Although our sample size was small, due to the very low prevalence of the disease, this is still one of the largest prospective studies in OT to date. Our study ended up recruiting a much higher number of female patients with OT. We believe this to be due to a number of factors including that there are more female patients with OT, that more female patients might be willing to volunteer for research participation, and that our patient recruitment was consecutive (not pre-specified by other variables like gender). The female preponderance of our sample might limit the generalizability of the results to males. Although there are some differences in the topographical distribution of alpha and theta rhythms between male and female subjects [29], these differences are unlikely to affect the EEG parameters assessed in our study.

Also, we did not perform the EEG and the EMG recordings simultaneously on all of our subjects due to technical difficulties.

Future studies using back averaging of EEG or measurement of cortical activity using magnetoencephalography could be done to further support normal cortical activity in OT.

## Conclusion

Our study provides evidence prospectively that EEG abnormalities are not typically seen in patients with OT. However, tremor artifact is extremely common, is usually consistent with EMG tremor frequency and should be considered when interpreting EEG findings in patients with OT.
